# From words to actions: systematic review of interventions to promote sexual and reproductive health of persons with disabilities in low- and middle-income countries

**DOI:** 10.1136/bmjgh-2020-002903

**Published:** 2020-10-15

**Authors:** Shaffa Hameed, Alexander Maddams, Hattie Lowe, Lowri Davies, Rajat Khosla, Tom Shakespeare

**Affiliations:** 1 International Centre for Evidence in Disability, London School of Hygiene & Tropical Medicine, London, UK; 2 Department of Reproductive Health and Research, WHO, Geneva, Switzerland

**Keywords:** systematic review, maternal health, public health, HIV, health services research

## Abstract

**Introduction:**

Persons with disabilities have the same sexual and reproductive health and rights (SRHR) as non-disabled persons. Yet they face numerous barriers in their access to sexual and reproductive health services and their rights are often not met. Evidence on SRHR for persons with disabilities is sparse, particularly evaluations of interventions demonstrating ‘what works.’ This systematic review assessed interventions to promote SRHR for persons with disabilities in low- and middle-income countries.

**Methods:**

We searched for qualitative, quantitative or mixed method observational studies representing primary research, published between 2010 and 2019, using MEDLINE, Embase, PubMed, Global Health and CINAHL Plus. Search strings were compiled for different elements of SRHR and for all forms of disability. 24,919 records were screened, leading to over 380 relevant papers, most of which were descriptive, focussing on needs and barriers to SRHR needs being fulfilled. Of the 33 full-text articles assessed for eligibility, 18 were included in the synthesis. All included studies were assessed for bias and quality of evidence, using STROBE (Strengthening the Reporting of Observational Studies in Epidemiology) and RATS (relevance, appropriateness, transparency andsoundness) tools. Among the 16 interventions (from 18 articles), 25% had low risk of bias, 31% had moderate risk of bias and 44% had high risk of bias. Data analysis used narrative synthesis; a method suited for systematic reviews with heterogeneous studies. We used Levesque healthcare access model to analyse the focus of interventions.

**Results:**

11 interventions were from upper middle-income settings; two from lower-income settings; only one operated in rural areas. Interventions addressed intellectual impairment (6), visual impairment (6), hearing impairment (4), mental health conditions (2) and physical impairments (2). Most interventions (15/16) focus on information provision and awareness raising. We could not identify any intervention promoting access to maternal health, family planning and contraception, or safe abortion for people with disabilities.

**Conclusion:**

This systematic review has highlighted stark gaps in evidence. More rigorous evaluations are needed.

**Key questions**

**What is already known?**

- Fifteen per cent of the global population—one billion people—are people with disabilities, with the same need for sexual and reproductive health and rights as non-disabled people.
- People with disabilities lack access to sexual and reproductive health (SRH) services and face violations of their human rights due to factors that range from inaccessible facilities, to communication barriers and negative attitudes.
- There is strong research and descriptive evidence documenting barriers and facilitators to SRH and rights (SRHR) attainment for people with disabilities.

**What are the new findings?**

- Relatively few studies evaluate interventions and their effectiveness in promoting SRHR for people with disabilities.
- Most interventions are set in upper-middle income contexts, urban areas and have tended to focus solely on information provision.
- Over half of these studies have high risk of bias as a result of poor study design.

**What do the new findings imply?**

- There is limited evidence to support the effectiveness of many interventions, despite promising intervention designs. More rigorous evaluations are needed.
- There is an urgent need to trial and evaluate more interventions in resource-poor settings, and expand on learning from high-income settings.
- Interventions need to go beyond information provision and health literacy, and address barriers to disabled people’s ability to seek, reach, pay and use services to achieve SRHR.

## Introduction

Research at the intersection of disability and sexual and reproductive health and rights (SRHR) is sparse—and the research and normative developments in the field of SRHR have often ignored people with disabilities and their specific sexual and reproductive health needs.^1–3^ There is often an attitude that disabled people are asexual.^3^ Evidence highlights wide-ranging and long-standing prejudices including myths: that impairments are incompatible with sexual desire and sexual activity; that disabled people cannot be parents; that disabled women do not experience sexual violence.

While disabled adults are less likely to be sexually active or in partnerships, research has shown that the disparity is not huge. In the USA, 50% of people with severe disabilities, 60% of people with non-severe disabilities and 68% of non-disabled people are married.^4^ Findings are similar in research in low- and middle-income countries (LMICs), across the different aspects of sexuality and reproduction. Jean-Francois Trani *et al*^5^ found that in urban areas of Sierra Leone, 58% of respondents with severe/very severe and 71% with mild/moderate disabilities had sexual intercourse in the previous year, compared with 92% of non-disabled respondents. Another study found 80% of 126 deaf people in Cameroon had been sexually active;^6^ while a study in Uganda found that 77% of women with disabilities had previously been pregnant.^7^

Although unintended pregnancy has long since been identified as a serious concern for women with disabilities (eg,^8^), estimates from the USA indicate that they were far less likely than women without disabilities to access family planning services.^9^ A similar US study showed that 30.2% of women with disabilities used female sterilisation compared with 18.8% of non-disabled women,^10^ highlighting the need to investigate issues of consent and knowledge gaps related to use of permanent methods for contraception. In a recent study in Nepal, women with severe impairment reported higher levels of physical and/or sexual, emotional, economic and in-law violence than women without a disability.^11^

Research on the intersection of disability and SRHR has been evolving. The lack of access to sexual and reproductive health (SRH) services experienced by people with disabilities is increasingly documented. Contributing factors include structural inaccessibility, communication barriers and negative attitudes from service providers (eg,^12^). Emphasis on rights-based research has illustrated further dimensions such as mistreatment and inadequacy in service delivery (eg,^3^). However, there is a lack of evidence underpinning interventions aimed at meeting SRH needs of persons with disability.^13^ Although evidence from high-income countries is insufficient, it appears that access to SRH is inequitable. The situation appears worse in LMICs, where the majority of the world’s billion persons with disabilities live, and which are therefore a priority for evidence synthesis and renewed efforts.

This systematic review helps fill that gap. Building on previous empirical and conceptual evidence on sexual and reproductive rights for people with disabilities (eg,^14–17^), this systematic review examines the following two questions: (1) what, if any, interventions are currently in place to promote sexual and reproductive health and rights of persons with disabilities in low- to middle-income countries? and (2) how effective are they?

## Methods

The systematic review adheres to the Preferred Reporting Items for Systematic Review and Meta-Analysis Protocols 2015 (PRISMA-P 2015) checklist—this is provided in online supplemental appendix A.^18^ The protocol is registered with PROSPERO, the international register for systematic reviews with the identification number CRD42019156379.

10.1136/bmjgh-2020-002903.supp1 Supplementary data

### Search strategy and selection criteria

Below we define the eligibility criteria used to select studies to be included in the systematic review:

- Any qualitative, quantitative or mixed method observational studies that represent original primary research: no restrictions were placed on study design.
- Studies involving persons with disabilities as recipients of intervention being investigated: the classification of disability reflect the WHO International Classification of Functioning, Disability and Health (ICF).^19^ Disability was classified as any form of physical, sensory, cognitive or psychosocial impairment associated with activity limitations/participation restriction.
- Studies that document interventions to promote SRHR for people with disabilities or report current measures used to address sexual and reproductive health needs of persons with disabilities: All forms of health measures from social policy to direct medical interventions were included to encompass the full scope of sexual and reproductive healthcare. Interventions may be targeted (available only for persons with disabilities) or inclusive (mainstream services available and accessible to persons with disabilities).
- Studies conducted in LMICs: we used the World Bank classification of countries by income.^20^
- Studies written in the English language.
- Studies published between 2010 and 2019/2020: searches were restricted to 2010 onwards to capture recent trends.

We excluded unpublished, non-peer-reviewed (grey) literature and did not back-reference (checking reference lists of eligible studies to identify more studies)—the implications of this are discussed later. Systematic reviews were included if they comprised studies that met the eligibility criteria. News articles, commentaries, policy documents and opinion pieces were excluded as they do not represent rigorous scientific study.

The following five databases were selected in consultation with the London School of Hygiene & Tropical Medicine (LSHTM) librarians: (1) MEDLINE via Ovid (1946 to present); (2) Embase via Ovid (1974 to present); (3) PubMed (1996 to present); (4) Global Health via Ovid (1973 to present) and (5) CINAHL (Cumulative Index of Nursing and Allied Health Literature) (1961 to present) Plus. The WHO Reproductive Health portal was initially selected but discarded as it does not allow any systematic searching (eg, use of Medical Subject Headings terms, Boolean operators).

To ensure comprehensive search terms across different aspects of SRHR, we compiled search strings for the themes below. These were identified by reviewing studies on similar topics, screening them to identify key terms overlooked by the team. Indicated below are some examples of terms under each theme (sample search strings are provided in full in the online supplemental appendix B):

10.1136/bmjgh-2020-002903.supp2 Supplementary data

- ‘Maternal Health’: includes antenatal, intrapartum, postnatal
- ‘Reproductive Health’ (RH): includes general RH services and programming, RH illnesses, urogenital disorders, menstrual health
- ‘Sexually Transmitted Infections’ (STIs): includes HIV/AIDS, prevention of mother-to-child transmission (PMTCT), testing, treatment
- ‘Comprehensive Sexuality Education’ (CSE): includes adolescent health, school-based interventions, peer education, information provision
- ‘Family planning and contraception’: includes emergency contraception, infertility
- ‘Abortion’: includes medical abortion, surgical abortion, miscarriage, abortion complications
- ‘Sexual violence’: includes gender-based violence, female genital mutilation
- ‘Sexual Health, Sexuality and Rights’: includes sexual dysfunction

Medical Subject Headings and keywords were used in combination with appropriate Boolean operators (‘AND’, ‘OR’ and ‘NOT’) and truncation to ensure the appropriate scope and relevancy when searching. These strings were refined, expanded and constricted to fit the scope of the review. Once finalised, the search built on MEDLINE was reviewed by the LSHTM Library Assistant. We were advised against using search terms to filter ‘interventions’, as these have not been tested rigorously and could exclude relevant articles.

All searches were run on the same day (23 November 2019) and by the same reviewer (SH) to limit variation. The final search strategy followed this formula: (SRHR terms) AND (disability terms) AND (LMIC terms) AND (time restriction 2010 to 2019/2020) AND (restriction by English language). The search yielded 39,306 results across the five databases. These were imported to EndNote X8 for de-duplication.

De-duplicated entries were imported to the web application Rayyan^21^ that allowed several team members to work on the same database, and tracked the decisions and progress made by each member. Reviewers first screened 10 articles to check consistency of decisions. Each then screened at least 6000 for relevance, first by title then abstract. A selection of excluded articles and all potentially included articles were independently screened by a second reviewer (SH) and any disagreement resolved by a third reviewer.

**Figure 1:**
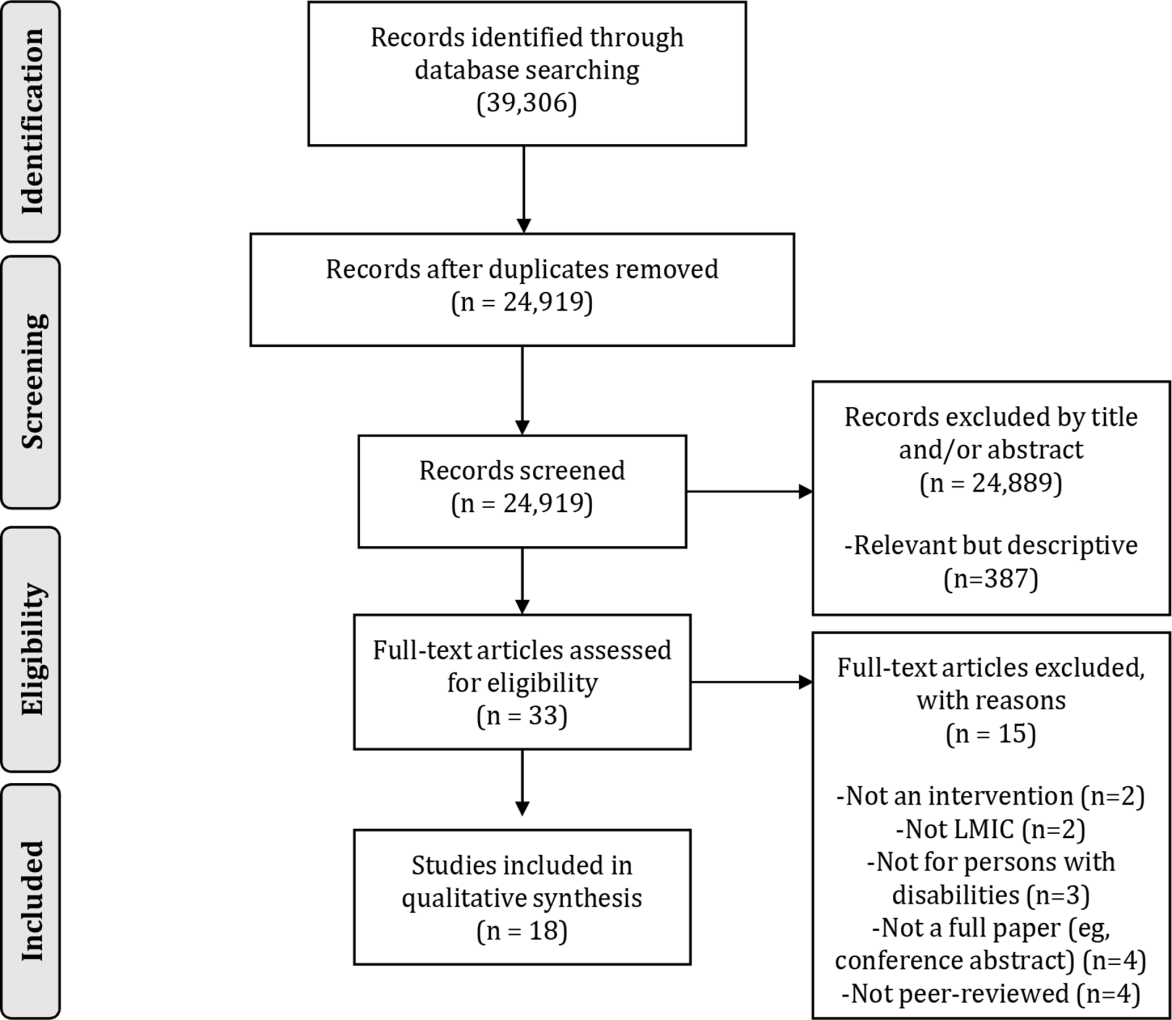
PRISMA (Preferred Reporting Items for Systematic Review and Meta-Analysis) of study selection process and search results. LMIC, low- and middle-income country.

Studies deemed potentially relevant were reviewed independently in full by the assigned reviewers. The final decision to include a study in the review was made by the entire team following discussion. Reasons for exclusion are documented in the PRISMA flowchart (figure 1).

Data from included studies were imported into Microsoft Excel for risk of bias assessment and analysis (described below). The following information were extracted: title, full citation, study setting (location, rural/urban), study population characteristics (age, gender, sample size, type of disability/impairment), study design and outcomes (data collection methods, analysis methods, limitations/confounders, main study findings and reported effectiveness of the intervention where possible).

### Data analysis

The main outcome of the study is a review of interventions currently used to promote SRHR of persons with disabilities. The secondary outcome is an assessment of effectiveness of these interventions, in order to investigate ‘what works.’ Several elements informed the data analysis: assessment of risk of bias, assessment of effectiveness, application of framework and finally the narrative synthesis. These are discussed in turn below.

#### Assessing risk of bias

Following data extraction, full texts of eligible articles were assessed by two reviewers (SH with either AM, HL or LD; differences were discussed) for risk of bias—a key step in systematic reviews that assesses the quality of evidence. Studies containing quantitative data were assessed using the Strengthening the Reporting of Observational Studies in Epidemiology (STROBE) checklist.^22^ These include criteria related to sampling methods (eg, representativeness, response rates), data collection (eg, validity and reliability of tool) and data analysis and interpretation (eg, confounders, statistical tests). Studies containing qualitative data were assessed using the guidance to authors in BioMed Central journals that examined relevance, appropriateness, transparency and soundness (RATS) in qualitative research.^23^ These include criteria related to study design (eg, appropriate methods), sampling (eg, detail given on sample characteristics and sampling method), data collection (eg, appropriate tools, bias) and data analysis and interpretation (eg, interpretation supported by evidence, reliability checks).

Study quality and overall confidence in the study findings were assessed based on how well they met the criteria. Given the variation in study designs, we avoided assigning numerical scores or applying a rigid cut-off criteria. Instead, studies were graded as having a low risk of bias when all or almost of the criteria were fulfilled, and those not fulfilled were thought unlikely to alter the conclusions of the study; a moderate risk of bias when some of the criteria were fulfilled, and those not fulfilled were thought unlikely to alter the conclusions of the study. Studies were categorised as having a high risk of bias when few or no criteria were fulfilled, and their inclusion were thought likely or very likely to alter the conclusions of the study.

#### Assessing effectiveness of interventions

The effectiveness assessment was undertaken in two steps, as done by Mikton and colleagues.^24^ First, we state the effectiveness reported in the study. Then, we take the study quality assessment into consideration, appraising whether or not there was no evidence, limited evidence or promising evidence to support that this intervention works.

#### Analytical framework

**Figure 2:**
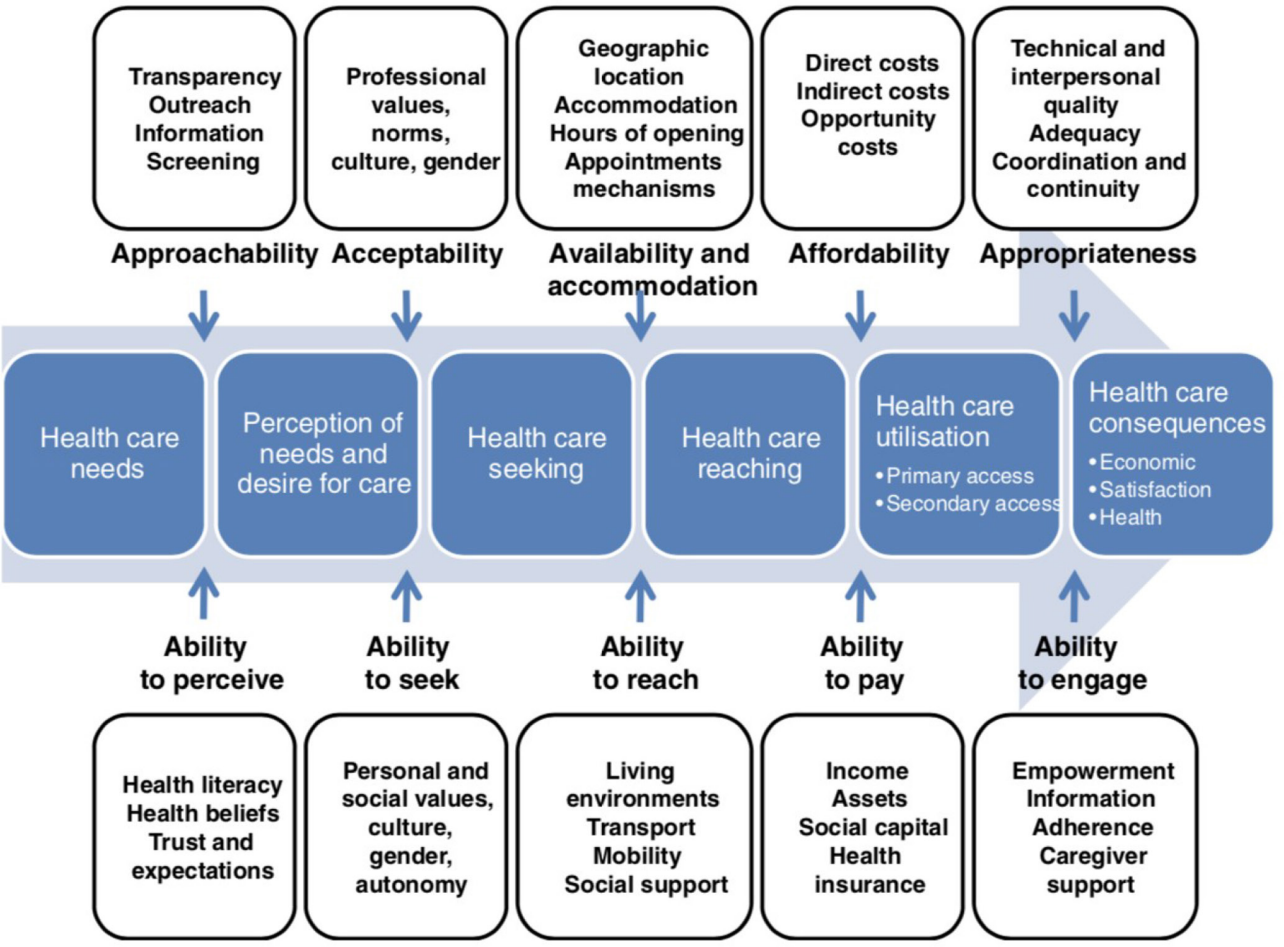
Levesque model for healthcare access.

For an analytical framework, we use the Levesque *et al* healthcare access model^25^ (see figure 2), chosen for two main reasons. First, this framework has proven utility in exploring access for people with disabilities as well as in capturing service provider accommodations for inclusion (eg,^26^). Second, this framework allowed us to identify what each intervention is targeting—whether it is addressing barriers related to supply or service provision (top row), and/or demand for services by people with disabilities (bottom row). Through this lens, we were able to capture patterns as well as highlight gaps.

#### Narrative synthesis

Our approach to data analysis was narrative synthesis, a method suited for systematic reviews with heterogeneous studies in terms of study design, interventions and outcomes. Using the guidance from Popay,^27^ our analysis moved iteratively between the four elements of narrative synthesis: (1) developing a theory of how the intervention works, considering why and for whom; (2) developing a preliminary synthesis of findings of included studies (among the suggested tools, we used contextual descriptions, groupings and clusters and tabulation); (3) exploring relationships in the data (through qualitative case descriptions and using conceptual diagrams); and (4) assessing the robustness of the synthesis (through quality appraisal techniques and critical reflection on the synthesis process).

##### Ethics

No ethical approval was required for this study.

##### Patient and public involvement

It was not appropriate to involve patients or the public in the design, or conduct, or reporting, or dissemination plans of our research.

## Results

The search described found over 380 entries on sexual and reproductive health and rights of persons with disabilities in LMICs. However, the vast majority of papers were descriptive, focussing on the needs of persons with disabilities and the barriers to those needs being fulfilled—these were subsequently excluded. Of the remaining 33 full-text articles assessed for eligibility, 15 did not meet the criteria.

### Study characteristics

Following the study selection process (figure 1), 18 articles were included in this systematic review. Of these, several papers concerned the same intervention, thus, a total of 16 different interventions are reviewed. Articles linked to the same intervention are discussed together, and discussions relate to interventions rather than articles. See table 1 for a summary of the characteristics of the included studies.

**Table 1:** Characteristics of included studies

Variable	Detail	Number of interventions	% of interventions
Study design	Qualitative	3	19
	Mixed methods	2	13
	Quantitative - cross-sectional survey	2	13
	Quantitative - case-control	1	6
	Quasi-experimental	3	19
	Quantitative - other	5	31
Risk of bias	Low risk of bias	5	31
	Moderate risk of bias	2	13
	High risk of bias	9	56
Income classification	Low income	2	13
	Lower-middle income	3	19
	Upper-middle income	11	69
Location	Rural	1	6
	Urban	8	50
	Mixed	7	44

Most studies were quantitative (n=11), with quasi-experimental, case-control or cross-sectional design. Five of the included 16 studies used qualitative methods, among which two were mixed methods.

Of the 16 interventions, some studies were assessed to have low risk of bias (31%, n=5), and two (31%) were identified to have moderate risk of bias. Most studies (56%, n=9) were graded as having a high risk of bias—that is, studies on these interventions did not fulfil most of the quality criteria. None of the studies were excluded based on study quality. Instead, this is taken into consideration in the effectiveness assessment (step 2), and subsequent analysis.

The majority of the interventions (69%, n=11) were set in upper middle-income countries—South Africa (n=3), Brazil (n=2), Iran (n=2)and one each in Ecuador, Turkey, Azerbaijan and the Philippines. Lower middle-income countries such as Tunisia, Nigeria and Indonesia each had one study. Only two interventions were based in low-income countries—one in Nepal and the other in Tanzania, indicating a stark lack of focus on the poorest contexts. Similarly, only one intervention was based in a rural setting, while half were in urban areas (50%, n=8) and the remaining in either mixed or semi-urban (44%, n=7).

Table 2 summarises the interventions by type of impairment. Several interventions were not specific to one type of impairment—thus, some studies were coded under more than one category. Thirty-eight per cent of the interventions were related to people with intellectual or cognitive impairments, and it was the same for those with visual impairments. In contrast, only two interventions (13%) each were related to people with mental health conditions or people with physical impairments. It may be that some interventions benefitting people with physical impairments were general but inclusive services (non-targeted, for example, ramps) and may not have been evaluated, while those targeting intellectual impairments may have been additional and targeted (eg, specific information material) and required evaluation before scaling up. Just three interventions (19%) targeted more than one type of impairment, and all three presented findings disaggregated by impartment type.

**Table 2:** Interventions by type of impairment

Type of impairment	Number of interventions	% of interventions
Physical impairment	2	13
Visual impairment	6	38
Hearing impairment	4	25
Intellectual/cognitive impairment	6	38
Mental disorders	2	13
Other impairment/functional limitations	1	6

The majority of the studies (n=11) involved data collected from people with disabilities, while some were based on data from carers or teachers. However, there was variation in disability definitions and assessment. Three used clinical definitions/assessments, one used the Washington Group questions on functional limitations and the remaining studies did not specify, which limits the application of findings. It is notable that among the 16 interventions, only five interventions (31%) involved Disabled People’s Organisations in intervention development, which would have strengthened suitability to context.

Although a number of different search strings were developed for the different components of SRHR, there were no eligible studies of interventions for many key themes, including maternal health, family planning, and abortion. As table 3 shows, 13% of the interventions promote general reproductive health (n=2) and sexual health (n=2) among people with disabilities, and their protection from STIs including HIV/AIDS (n=2). 19% of the interventions (n=3) relate to protection from sexual violence. Nearly half of the included interventions focus on CSE and information provision in and out of schools, though some interventions were coded both as CSE (for delivery method) and content (e.g., violence).

**Table 3:** Interventions and evidence quality by SRHR theme

SRHR theme	Interventions		Evidence						
	n	%							
Maternal health (incl. antenatal, intrapartum, postnatal)	0	0	No evidence						
Reproductive health (incl. general RH services and programming, RH illnesses, urogenital disorders, menstruation)	2	13	Limited			Promising			
STIs (incl. HIV/AIDS, PMTCT, testing, treatment)	2	13	Limited			Limited			
CSE (incl. adolescent health, school-based, peer education, information provision)	7	44	L	L	L	L	P	P	P
Family planning and contraception (incl. emergency contraception, infertility)	0	0	No evidence						
Abortion (incl. medical, surgical, miscarriage, complications)	0	0	No evidence						
Sexual violence (incl. GBV, FGM)	3	19	Limited		Limited		Promising		
Sexual health, sexuality and rights (incl. sexual dysfunction)	2	13	Limited			Promising			

CSE, comprehensive sexuality education; FGM, female genital mutilation; GBV, gender-based violence; incl., including; L, limited; P, promising; PMTCT, prevention of mother-to-child transmission; RH, reproductive health; SRHR, sexual and reproductive health and rights; STIs, sexually transmitted infections.

Table 4 summarises the data extracted from the studies, displaying details of intervention, targeted impairment type, SRHR theme, effectiveness before and after considering risk of bias in the evidence and the Levesque dimension of access each intervention targets. Some elements of our interpretation based on these processes are also included. For example, if an intervention was claimed to be effective, but the study making this claim had a high risk of bias, we interpret that there is limited evidence to support that this intervention ‘works’. We then examine the Levesque dimensions to interpret whether the intervention is promising or limited in terms of wider application potential. Combined, each row highlights strengths and weakness of both evidence and intervention, which then frames the subsequent discussion.

**Table 4:** Summary of data extracted

Author (s)	Intervention	Setting	Study design	Type of impairment(s)	(2a) Effectiveness assessment without considering risk of bias	(1) Risk of bias	(2b) Effectiveness assessment with risk of bias	(1+2) Evidence to support this intervention ‘works’	(3a) Levesque: patient dimension	(3b) Levesque: service dimension	(3 overall) Intervention	SRHR theme
Fiander and Vanneste (2012)^28^	Provides cash to meet transport cost of attending obstetric fistulae surgery	Tanzania (LIC)	Quantitative	Other functional limitations	Effective	High	Unclear	Insufficient evidence	Ability to pay	Affordability	Promising intervention: potential application (other impairments, other topics)	RH
Ghaderi *et al* (2017)^44^	Provides self-encouragement sessions (10×90 mins) to women with physical impairments	Iran (upper MIC)	Quasi-experimental	Physical	Effective	High	Unclear	Insufficient evidence	Ability to perceive	(None)	Unclear wider application or impact	Sexuality
Hanass-Hancock *et al* (2014)^33^	A tool, Teachers’ Sexuality Education Questionnaire (TSE-Q), to capture attitudes, needs and experiences of teaching CSE to disabled children	South Africa (upper MIC)	Mixed methods	Visual, Hearing, Intellectual	Effective	Moderate	Effective	Promising evidence	Ability to perceive (anticipated)	Approachability Acceptability	Promising intervention: potential application (other impairments, other contexts)	CSE
Hanass-Hancock *et al* (2018a)^34^	Breaking the Silence approach to deliver CSE to children with intellectual impairment: educators find it effective	South Africa (upper MIC)	Qualitative	Intellectual	Effective	Moderate	Effective	Promising evidence	Ability to perceive	Approachability Acceptability	Promising intervention: potential application (other contexts)	CSE
Hanass-Hancock *et al* (2018b)^35^	Breaking the silence approach to deliver CSE to children with intellectual impairment: need to address contextual factors	South Africa (upper MIC)	Qualitative	Intellectual		Low						
Mdikana and Phasha (2018)^42^	School-based support teams that provide information support to intellectually disabled students at risk of sexual abuse	South Africa (upper MIC)	Qualitative	Intellectual	Effective	High	Unclear	Insufficient evidence	Ability to perceive reach (anecdotal)	Approachability	Promising intervention: potential application (other impairments)	Violence
Nakhli *et al* (2014)^45^	Arabic version of a tool to screen people with schizophrenia for sexual dysfunction	Tunisia (lower MIC)	Quantitative	Mental health	Effective	Low	Effective	Promising evidence	Ability to perceive	Approachability	Promising intervention: potential application (other contexts)	Sexual health
Neherta *et al* (2019)^43^	Provides information to mothers of intellectually impaired children about violence	Indonesia (lower MIC)	Quasi-experimental	Intellectual	Effective	High	Unclear	Insufficient evidence	Ability to perceive	(None)	Intervention unclear, limited scope	Violence (and CSE)
Oliveira *et al* (2016)^31^	Package to provide STI information to people with visual impairment	Brazil (upper MIC)	Quantitative	Visual	Effective	High	Unclear	Insufficient evidence	Ability to perceive	(None)	Unclear utility of their intervention design	STIs (and CSE)
Oliveira *et al* (2018)^37^	Information package on RH and fertility delivered in Braille for people with visual impairment	Brazil (upper MIC)	Quant - cross-sectional	Visual	Positive	High	Unclear	Limited evidence	Ability to perceive	(None)	Intervention limited in scope and application	CSE (and RH)
Robles-Bykbaev *et al* (2019)^36^	Web-based platform where SRH information is delivered through sign language	Ecuador (upper MIC)	Quantitative	Hearing	Effective	Low	Effective	Promising evidence	Ability to perceive	Approachability	Promising intervention: potential application (other contexts, other topics)	CSE (and RH)
Salahi *et al* (2018)^41^	Woman Abuse Screening Tool (WAST), is assessed to capture IPV among people with mental health disorders	Iran (upper MIC)	Quant - cross-sectional	Mental health	Effective	Low	Effective	Promising evidence	Ability to perceive	Approachability	Promising intervention: potential application (other contexts)	Violence
Wilbur and Bright *et al* (2018)^29^	Steps in developing a behaviour change intervention for people with intellectual disabilities on MHM	Nepal	Qualitative	Intellectual	Effective	Moderate	Effective	Promising evidence	Ability to perceive seek	Acceptability	Promising intervention: potential application (other contexts)	RH
Wilbur *et al* (2019)^46^	Intervention for people with intellectual impairments on MHM	Nepal (LIC)	Mixed methods			Low						
Yildiz *et al* (2017)^39^	Sexuality Education Program for Mothers of Young Adults with Intellectual Disabilities (SEPID) (5×120 mins)	Turkey (upper MIC)	Quant - case-control	Intellectual	Effective	Moderate	Unclear	Limited evidence	Ability to perceive	(None)	Intervention limited in scope and application	CSE
Devine *et al* (2017)^40^	Participant Action Groups to improve self-confidence in SRH seeking, SRHR knowledge, peer support	Philippines (upper MIC)	Qualitative	Visual; Hearing; Physical	Effective	High	Unclear	Limited evidence	Ability to perceive seek engage	(None)	Promising intervention: potential application (other contexts)	CSE (and RH, SH violence)
Doherty *et al* (2016)^32^	A multi-pronged intervention including accessible material provision, peer education and condom programme to disabled students	Nigeria (lower MIC)	Quantitative	Visual; Hearing	Effective	High	Unclear	Limited evidence	Ability to: perceive seek	Approachability Acceptability	Promising intervention: potential application (other topics)	STIs (and CSE)
Aval *et al* (2019)^38^	Provides information to VI women (includes Q&As and models) on RH, STIs, motherhood, menstruation.	Azerbaijan (upper MIC)	Quasi-experimental	Visual	Effective	High	Unclear	Limited evidence	Ability to perceive	(None)	Intervention limited in scope and application	CSE (and RH)

CSE, comprehensive sexuality education; IPV, Intimate Partner Violence; LIC, low-income country; MHM, menstrual hygiene management; MIC, middle-income country; Q&As, questions and answers; RH, reproductive health; SH, sexual health; SRH, sexual and reproductive health; SRHR, sexual and reproductive health and rights; STI, sexually transmitted infection; VI women, visually impaired women.

### Reproductive health

In this systematic review, this theme includes general RH services and programming, RH illnesses, urogenital disorders, and menstrual hygiene management (MHM). Two interventions were identified—one promoting access to MHM and the other on access to obstetric fistulae surgery.

**Fiander and Vanneste (2012**) report on an intervention that used community-based ‘ambassadors’ to provide transport money to patients who needed surgery for obstetric fistulae and cleft lip in Tanzania.^28^ In 2010, 239 patients attended 129 obstetric fistula surgeries—four times the scheme target and resulted in a 65% increase in the fistula operations performed. However, this study had a high risk of bias: reviewers note that there was little evidence that potential confounders or intervening factors that may have contributed to the increase in surgeries were considered in interpreting the findings. The intervention may have promise in that it responds directly to patients’ financial barriers (people’s ability to pay, service affordability), and could contribute to improvements in healthcare utilisation. This intervention had potential for wider application, beyond RH surgeries and people with other impairments, though a rigorous evaluation is first needed.

**Wilbur and Bright *et al* (2018**) documented the development of an intervention to improve menstrual hygiene management for women with intellectual disabilities in Nepal^29^ and **Wilbur *et al* (2019**) reported on a feasibility study of this intervention conducted with 10 women with intellectual impairment and their 8 carers.^30^ Pre- and post-survey and process monitoring found that the Bishesta campaign was acceptable, was delivered with fidelity and improved target behaviours. Although some indicators of quality were not reported in Wilbur and Bright *et al* (2018)^29^ because it was a methodological paper, the feasibility study (Wilbur *et al* 2019) was assessed to have low risk of bias, indicating promising evidence that this intervention works. The intervention has promise as it promotes intellectually impaired people’s ability to perceive and seek support services, while also promoting acceptability of services/products related to their menstrual hygiene. Further testing in other settings would confirm wider application.

### Sexually transmitted infections

This theme includes HIV/AIDS interventions such as PMTCT (prevention of mother-to-child transmission), testing and treatment. Two interventions were identified for this theme, both of which were assessed to have high risks of bias, indicating limited evidence in this theme.

**Oliveira *et al* (2016**) conducted a validation study of an information package with content experts reviewing educational materials (a rhyming approach adapted from breast feeding promotion material) on STIs for visually impaired people in Brazil.^31^ They reported positive results, with some adaptations needed to ensure accuracy of STI information. Reviewers assessed this study to have a high risk of bias, linked to inadequate sampling technique, unsuitable data collection and analysis methods and interpretation without sufficient detail to convey reliability or rigour. The intervention targeted visually impaired people’s health literacy (ability to perceive need for care)—this is of great importance. However, there was insufficient evidence to indicate that this approach works, and wider application was unclear.

**Doherty *et al* (2016**) reported on a situational analysis of a 2-year large-scale HIV prevention intervention for in-school children with impairment in Nigeria.^32^ They reported that the intervention led to increased SRH knowledge among hearing and visually impaired students, improved health seeking behaviours and access to HIV services, reporting an 80% increase in uptake of HIV counselling and testing services. This study was assessed to have a high risk of bias because of insufficient detail on methodology (data collection methods, analytical approach, sample characteristics) of the end-of-project evaluation that presumably links the intervention activities to increase in uptake. The intervention seems promising given that it targets disabled people’s ability to perceive and seek SRH services, as well as approachability and acceptability of these services through wide stakeholder and community engagement.

### Comprehensive sexuality education

For this review, we categorised school-based interventions, peer education, information provision in and out of schools as part of CSE. Of the seven interventions, three were found to have promising evidence, while the remaining had limited evidence supporting their effectiveness. All interventions promoted disabled people’s ability to perceive the need for services, with only one intervention (by Devine *et al* 2017) also promoting their ability to seek and engage.

**Hanass-Hancock *et al* (2014**) reported on the tool (Teachers’ Sexuality Education Questionnaire, TSE-Q) to assess teachers’ needs, knowledge, attitude, practice and self-efficacy in delivering sexuality education to disabled children in South Africa.^33^ They concluded that this tool has cross-cultural validity, working well to capture educators’ attitudes, practice, self-efficacy and perceived norms, although further work was needed to better capture educators’ knowledge. This study was assessed to have a moderate risk of bias because of limited detail on sample characteristics at individual level. However, this was not expected to significantly change the outcome of the study. This is a promising intervention in that it targets approachability and acceptability of CSE for children with sensory and intellectual impairments and could potentially be applied to other impairment types and in other contexts.

**Hanass-Hancock *et al* (2018a**) **and (2018b)** were two papers derived from the qualitative component of the formative evaluation on the CSE intervention Breaking the Silence implemented in South Africa, in eight schools for learners with special educational needs. Of these, the former study (2018a) explored educator’s perspectives, finding that the intervention can be delivered by educators after a 3-day training, and that it was effective in improving educator’s skills in delivering CSE to intellectually impaired children.^34^ However, this study was assessed to have moderate risk of bias, as there was little detail on how the sample of educators were recruited (eg, whether it was purposive to capture diversity of opinion). This was not expected to significantly alter the conclusions of the study.

**Hanass-Hancock *et al* (2018b**) explored contextual factors that inhibit children with disabilities in getting access to CSE, identifying factors including physical and information access barriers; negative social attitudes, educator and parent discomfort in discussing some topics (eg, genitalia).^35^ Crucially, they report that a critical mass of staff, including management, needs to be trained for greater effectiveness. This study was assessed to have low risk of bias, indicating promising evidence that this intervention is effective. The intervention targets approachability and acceptability of providing CSE to children with disabilities and shows great promise in terms of wider application to other contexts.

**Robles-Bykbaev *et al* (2019**) reported on an intervention for deaf women in Ecuador, creating a web-based platform where SRH information was conveyed through sign language as well as allow users to interact.^36^ They reported positive feedback and acceptability of the platform among people working with deaf women (mainly deaf educators) as did educators, clinicians and others. Reviewers initially differed in risk of bias assessments: some felt that the purposive sampling approach could have created bias, while others felt that this would not have significantly changed the outcome of the study. The intervention targeted approachability of SRH services for hearing impaired people through engagement with service providers and showed promise in wider application to deliver other SRHR topics and in other settings.

**Oliveira *et al* (2018**) evaluated a Braille manual delivering RH information over 3 to 15 days to women with visual impairment in Brazil.^37^ Through pre-intervention and post-intervention testing, they concluded that the manual improved participants’ knowledge, regardless of sociodemographic characteristics and congenital or acquired blindness. Reviewers assessed this study to have a high risk of bias as a result of their sampling method, sample size and lack of a control group that better determine the effectiveness of the intervention.

**Aval** ***et al*** **(2019)**, too, reported on an educational package delivering RH information (over 2 days) to visually impaired women through Braille in Azerbaijan.^38^ Following pre-intervention and post-intervention tests, they reported a statistically significant improvement in knowledge regarding menstrual health, RH, STIs and pregnancy care. This study was assessed to have a high risk of bias given that possible confounders (eg, small sample size (n=26), and difficulties recruiting sample) did not seem to be considered in data interpretation. Neither of these interventions addressed any barriers in service provision and both seem limited in scope.

**Yildiz and Cavkaytar (2017**) conducted a case-control study of a Sexuality Education Program for Mothers of Young Adults with Disability (SEPID), an educational programme for families on how to communicate with their children about sexuality education in Turkey.^39^ The results showed that SEPID changed attitudes of mothers towards sexuality education for their children and improved perception of social support. However, this study was assessed to have moderate risk of bias as the sample recruitment may have had elements of self-selection. The intervention did not address any barriers from the supply/service provision dimension and was limited in scope.

**Devine *et al* (2017**) reported on a qualitative study assessing the effectiveness of a 3-year programme of participatory action research (including peer action groups) to improve access to quality SRH for women with disabilities in Philippines.^40^ They reported improvements in self-confidence in SRH seeking, SRHR knowledge and social participation among peers. Whether or not these effects are sustained over time was shaped by personal and community level factors. This study had a high risk of bias stemming from various confounders that were not reported in the data interpretation including conflicts of interest (intervention coordinator conducted the evaluation interviews), presumed intervention effect (participants were asked for ‘stories of change’) and insufficient detail on sample recruitment. This intervention held promise because it promoted disabled people’s ability to perceive SRHR needs, to seek services and to engage with services to demand adequacy. It was one of the two interventions in this review with direct links to healthcare utilisation, and a rigorous evaluation is needed to support its effectiveness.

### Sexual violence

The theme ‘sexual violence’, including gender-based violence and female genital mutilation, had one intervention with promising evidence and two with limited evidence.

**Salahi *et al* (2018**) assessed validity of an intimate partner violence screening tool in women with mental health conditions at a psychiatric hospital in Iran.^41^ They found that the Farsi version of the Women Abuse Screening Tool (WAST) (and the WAST-Short Form) was easy to implement, suitable for initial screening in busy settings and correlated well with prevalence from the reference standard Conflict Tactics Scale-2. This study was assessed to have low risk of bias, indicating promising evidence to support this intervention’s effectiveness. The intervention, too, has promise because it extends application of a mainstream tool to women with mental health conditions, promoting people’s ability to perceive their SRHR needs as well as enhancing approachability of such services.

**Mdikana and Phasha (2012**) examined the functionality of school-based support teams (SBSTs) providing information support to children with intellectual disabilities identified to be at risk of or experiencing sexual abuse in South Africa.^42^ The study concluded that the SBSTs were functioning well despite receiving little support from the district-level teams, mentioning instances where SBSTs had gone beyond their role to provide counselling, accompanying the child to facility, helping with reporting to authorities. However, reviewers noted that this study had a high risk of bias relating to the use of convenience sampling, having a biased sample (only SBSTs were included)and insufficient detail on data collection tool and analysis. There may be promise to this approach involving school-based structures to respond to sexual violence experienced by intellectually impaired children. It promotes people’s ability to perceive the need for care as well as approachability of services. However, a more rigorous evaluation of functionality, barriers and facilitators, incorporating multiple perspectives, is needed.

**Neherta *et al* (2014**) reported on an intervention (via slides, videos, discussions) to improve mothers’ knowledge on sexual violence prevention for children with intellectual impairments in Indonesia.^43^ Using pre-interventions and post-interventions questionnaires, they reported improvements to mothers’ knowledge and attitudes. However, this study had a high risk of bias as several crucial quality criteria were not fulfilled. These included a lack of detail on sample characteristics, response rate, sampling method, whether participants were protected from negative or non-response (all mothers of children receiving care at the facility conducting the study), confounders and intervening factors that may have influenced participant attitudes and knowledge. There was little detail about the intervention, which targeted people’s ability to perceive SRHR needs and did not address any barriers from the supply/service provision dimension, making it limited in scope and application.

### Sexual health, sexuality and rights

For this review, we included sexual health, sexuality and sexual dysfunction under one theme, and identified two interventions—one with promising evidence and the other limited.

**Ghaderi *et al* (2017**) reported on the effectiveness of a self-encouragement skills training to improve genital self-image in women with physical impairments in Iran.^44^ After conducting a quasi-experimental study with pre-test and post-test (with 25 women each in intervention and control arms) using the Female Genital Self-Image Scale, they reported improvements to participants’ genital self-image by 61%. However, this study was assessed to have a high risk of bias because of limited detail on control matching, sampling, disability assessments, and in acknowledging intervening factors that may have influenced participant responses. There was little detail about the intervention, which targeted people’s ability to perceive SRHR needs and did not address any barriers from the supply/service provision dimension, making it limited in scope and application.

**Nakhli *et al* (2014)** assessed the validity of Arabic version of the Arizona Sexual Experiences Scale (ASEX) that is used to assess sexual dysfunction among patients with schizophrenia in Tunisia.^45^ Administering the translated tool to patients with schizophrenia (n=100), they found it had ‘highly acceptable’ reliability and validity. The study was assessed to have low risk of bias despite limited information being provided about the sample: it was unlikely that this would alter the outcome of the study. This intervention held promise as the translated tool will extend the use of the ASEX to assess sexual dysfunction among people with schizophrenia in Arab speaking contexts, promoting people’s ability to perceive the need for care, as well as approachability of services.

### Dimensions of access

**Figure 3:**
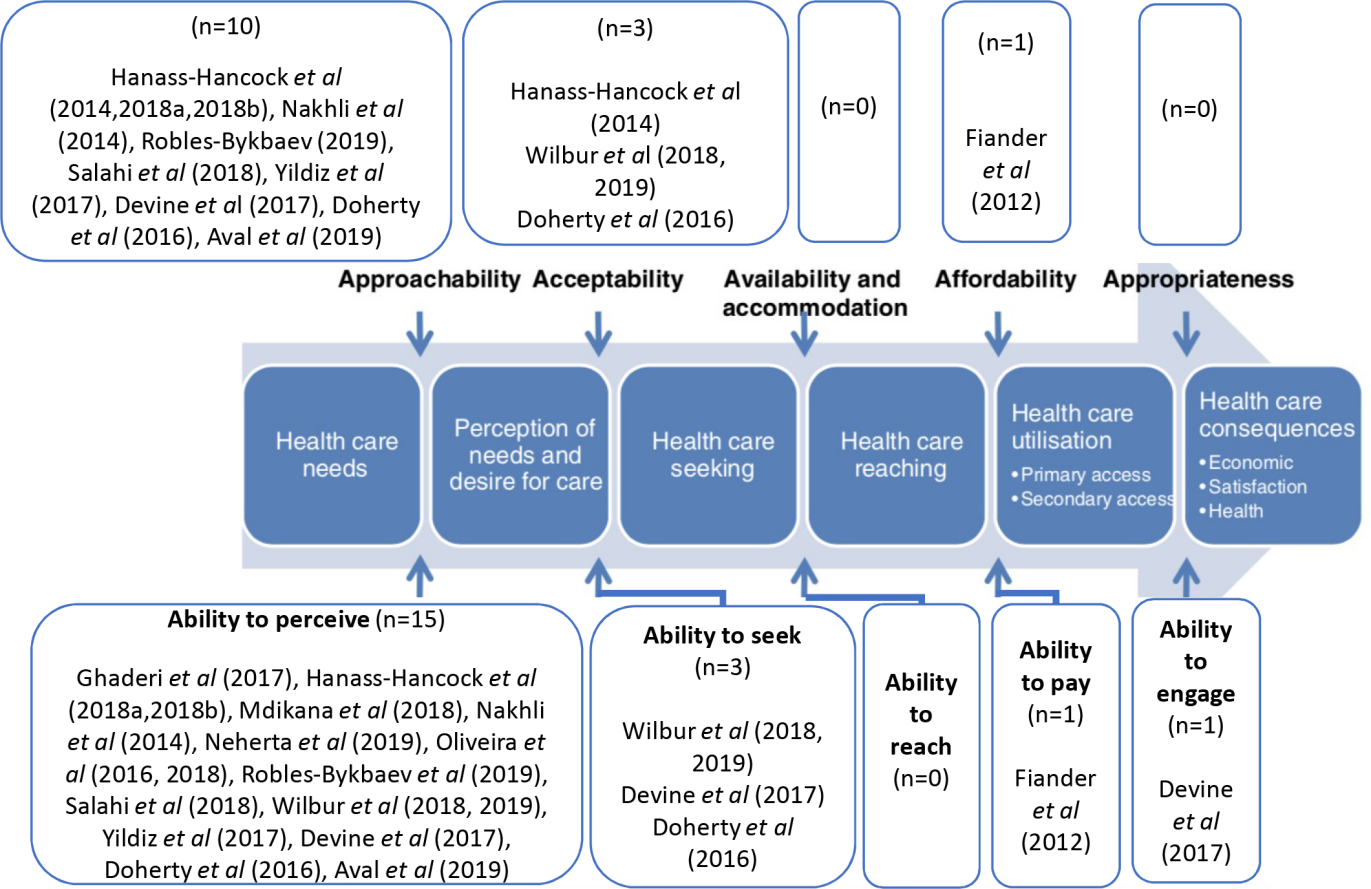
Interventions mapped on Levesque access model.

Figure 3 below demonstrates the dimensions of Levesque framework of access^25^ targeted by the interventions in this review.

Evidently, there was a disproportionate focus on promoting people’s ability to perceive their SRHR needs: 15 of the 16 interventions in this systematic review focus on this. Though an important entry step in the pathway to achieving optimum SRHR, very few interventions went beyond information provision. Wilbur *et al* (2018, 2019)^29 46^ provided information on MHM to women with intellectual impairments as well as promoting their autonomy. Devine *et al* (2017)^40^ and Doherty *et al* (2018),^32^ despite limited evidence to support its effectiveness, designed interventions that promoted access through multiple domains. Fiander *et al* (2012),^28^ though also with limited evidence to confirm its effectiveness, targeted disabled people’s ability to pay for services to achieve better SRH.

Using the Levesque framework,^25^ we have highlighted that interventions for people with disabilities promoting their SRHR have rarely gone beyond raising awareness.

## Discussion

This systematic review has demonstrated that interventions to promote SRHR of people with disabilities are limited in number and scope. While there were nearly 400 studies that documented disabled people’s SRHR needs, barriers and facilitators, we identified only 18 where research was translated into interventions. Furthermore, the majority of these were set in upper middle-income countries, and only one set in a rural area, indicating neglect of resource-poor settings.

Despite in-depth and dedicated search strategies on the different aspects of SRHR, we were unable to identify any studies on interventions related to promoting access to maternal health, family planning and contraception and safe abortion for people with disabilities. This is disconcerting because barriers to accessing SRHR services have been well documented. Among the 400 papers excluded from this review was a study indicating information and communication barriers to family planning experienced by deaf women in Ghana. As one respondent said “It is true that deaf people do not have enough knowledge on pregnancy and engage in sex without protection. […] You see, the TVs and newspapers are not accessible to deaf people, so how can deaf people understand this information from doctors and nurses? Our girls are always getting pregnant.” (female age 44)^47^

The lack of coverage may be a result of publication bias (discussed below) or it may also be indicative of deep-seated pervasive belief that people with disabilities are asexual^48^ or desexualised, separating “sexuality from disabled bodies, making it irrelevant to and incompatible with them because […] disability is believed to lead to sexual incapacity”.^49^

The majority of the interventions that were identified focussed on information provision, rather than trialling innovations in service delivery or barrier removal. Information provision seems the easiest step in the path towards full SRHR attainment, particularly if subsequent movement towards seeking and using services is not enabled. Little is being done to address barriers to availability, accommodation and appropriateness of services. In order to advance along the access pathway from healthcare seeking to healthcare delivery and utilisation, more investments are needed to evaluate current accommodation measures and quality and adequacy of care received by people with disabilities.

Two sorts of approach are necessary: first, removal of barriers so that mainstream services could be inclusive of all; second, development of targeted interventions—such as the Bishesta campaign^29 46^—to address the additional needs of some persons with disabilities.

It is important to acknowledge that this review may have had publication and ‘innovation’ bias. Targeted interventions (eg, for those with intellectual impairments) may require specific material or products to be developed, which would make them more likely to be evaluated and published. By contrast, non-targeted interventions (eg, installing a ramp at a maternal health facility entrance) may not be perceived ‘innovative’ enough to warrant an evaluation or academic publication, and thus would not have been captured in this systematic review. Similarly, we acknowledge that many activities by government or development agencies may be documented only in grey literature (eg, reports), which are not captured in this review but could be an important next step to expand this work.

This systematic review has highlighted important shortcomings in the work towards Sustainable Development Goals (SDG) related to SRH. SDG Goals 3.7 and 5.6, which seeks to ensure health and well-being for all, must involve actions and interventions to promote access to SRH services for people with disabilities.^50^ This review has highlighted a complete lack of evidence on interventions promoting disabled people’s access to maternal health, family planning and safe abortion services; and limited evidence on interventions related to general reproductive health, and protection from STIs. There is slightly better progress on SDG 4 on ensuring inclusive and quality education for all,^50^ given the increasing and promising evidence on interventions promoting access to inclusive sexuality education. However, for SDG 5 promoting gender equality and the empowerment of women and girls,^50^ there is limited evidence to support interventions that protect women with disabilities from sexual and gender-based violence. The SDG ambition is echoed by the Convention on the Rights of Persons with Disabilities which emphasises the importance of delivering accessible, high-quality health services to people with disabilities without discrimination in Article 25, Health, as well as Article 23, Respect for Home and Family.^51^ However, by these standards too, there is much work to be done.

## Conclusion

This systematic review has highlighted stark gaps in evidence about interventions to promote SRHR for persons with disabilities. Disabled people’s organisations should be consulted and involved in barrier removal and intervention development activities. More rigorous evaluations are needed. Many interventions included in this review had promise, but their effectiveness could not be confirmed due to limited evidence from poorly designed evaluations. Studies need to use robust methodologies, consistent definitions of disability and to be trialled in resource-poor settings. More actions, and more implementation research, particularly impact evaluations, are urgently required to promote SRHR for people with disabilities in LMICs.
