# Supplementary material for: From words to actions: systematic review of interventions to promote sexual and reproductive health of persons with disabilities in low- and middle-income countries
Source: BMJ Glob Health. 2020 Oct 15;5(10):e002903. doi: 10.1136/bmjgh-2020-002903 (PMC7566436; doi:10.1136/bmjgh-2020-002903)
Supplement: Supplementary data [file bmjgh-2020-002903supp002.pdf]

| LMICs | |
|---|---|
| 1 | developing countr*[MH] OR developing countr*[TIAB] OR developing nation*[TIAB] or developing world[TIAB] |
| 2 | least developed countr*[TIAB] OR least developed nation*[TIAB] OR least developed world[TIAB] OR least-developed countr*[TIAB] OR least-developed nation*[TIAB] OR less-developed countr* OR less-developed nation*[TIAB] OR less developed countr*[TIAB] OR less developed nation*[TIAB] |
| 3 | under-developed countr*[TIAB] OR under developed countr*[TIAB] OR underdeveloped countr*[TIAB] OR under-developed nation*[TIAB] OR under developed nation*[TIAB] OR underdeveloped nation*[TIAB] OR under-developed world[TIAB] OR under developed world[TIAB] OR underdeveloped world[TIAB] OR under-developed econom*[TIAB] OR under developed econom*[TIAB] OR underdeveloped econom*[TIAB] |
| 4 | third world countr*[TIAB] OR third world nation*[TIAB] OR third-world countr*[TIAB] OR third-world nation*[TIAB] |
| 5 | low- and middle-income countr*[TIAB] OR low and middle income countr*[TIAB] OR low- and middle-income nation*[TIAB] OR low and middle income nation*[TIAB] OR low- and middle-income world[TIAB] OR low and middle income world[TIAB] OR low- and middle-income econom*[TIAB] OR low and middle income econom*[TIAB] OR low income countr*[TIAB] OR middle income countr*[TIAB] OR low-income countr*[TIAB] OR middle-income countr*[TIAB]OR low income nation*[TIAB] OR middle income nation*[TIAB] OR low-income nation*[TIAB] OR middle-income nation*[TIAB] OR low income world[TIAB] OR middle income world[TIAB] OR low-income world[TIAB] OR middle-income world[TIAB] OR low income econom*[TIAB] OR middle income econom*[TIAB] OR low-income econom*[TIAB] OR middle-income econom*[TIAB] |
| 6 | LIC[TIAB] OR LICs[TIAB] OR MIC[TIAB] OR MICs[TIAB] OR LMIC[TIAB] OR LMICs[TIAB] OR LAMIC[TIAB] OR LAMICs[TIAB] OR LAMI countr*[TIAB] |
| 7 | Transitional countr*[TIAB] OR Transitional econom*[TIAB] OR Transition countr*[TIAB] OR Transition econom*[TIAB] |
| 8 | Asia[MH] OR Africa[MH] OR South America[MH] OR Caribbean region[MH] OR Central America[MH] |
| 9 | Afghanistan[TIAB] OR Albania[TIAB] OR Algeria[TIAB] OR American Samoa[TIAB] OR Angola[TIAB] OR Antigua[TIAB] OR Barbuda[TIAB] OR Argentina[TIAB] OR Armenia[TIAB] OR Azerbaijan[TIAB] OR Bangladesh[TIAB] OR Belarus[TIAB] OR Byelarus[TIAB] OR Byelorussia[TIAB] OR Belorussia[TIAB] OR Belize[TIAB] OR Benin[TIAB] OR Bhutan[TIAB] OR Bolivia[TIAB] OR Bosnia[TIAB] OR Herzegovina[TIAB] OR Hercegovina[TIAB] OR Bosnia-Herzegovina[TIAB] OR Bosnia-Hercegovina[TIAB] OR Botswana[TIAB] OR Brazil[TIAB] OR Brasil[TIAB] OR Bulgaria[TIAB] OR Burkina[TIAB] OR Upper Volta[TIAB] OR Burundi[TIAB] OR Urundi[TIAB] OR Cambodia[TIAB] OR Republic of Kampuchea[TIAB] OR Cameroon[TIAB] OR Cameroons[TIAB] OR Cape Verde[TIAB] OR Central African Republic[TIAB] OR Chad[TIAB] OR Chile[TIAB] OR China[TIAB] OR Colombia[TIAB] OR Comoros[TIAB] OR Comoro Islands[TIAB] OR Comores[TIAB] OR Congo[TIAB] OR DRC[TIAB] OR Zaire[TIAB] OR Costa Rica[TIAB] OR Cote d'Ivoire[TIAB] OR Ivory Coast[TIAB] OR Cuba[TIAB] OR Djibouti[TIAB] OR Obock[TIAB] OR French Somaliland[TIAB] OR Dominica[TIAB] OR Dominican Republic[TIAB] OR Ecuador[TIAB] OR Egypt[TIAB] OR United Arab Republic[TIAB] OR El Salvador[TIAB] OR Eritrea[TIAB] OR Ethiopia[TIAB] OR Fiji[TIAB] OR Gabon[TIAB] OR Gabonese Republic[TIAB] OR Gambia[TIAB] OR Georgia[TIAB] OR Ghana[TIAB] OR Gold Coast[TIAB] OR Grenada[TIAB] OR Guatemala[TIAB] OR Guinea[TIAB] OR Guinea-Bissau[TIAB] OR Guiana[TIAB] OR Guyana[TIAB] OR Haiti[TIAB] OR Honduras[TIAB] OR India[TIAB] OR Indonesia[TIAB] OR Iran[TIAB] OR Iraq[TIAB] OR Jamaica[TIAB] OR Jordan[TIAB] OR Kazakhstan[TIAB] OR Kenya[TIAB] OR Kiribati[TIAB] OR Republic of Korea[TIAB] OR North Korea[TIAB] OR DPRK[TIAB] OR Kosovo[TIAB] OR Kyrgyzstan[TIAB] OR Kirghizstan[TIAB] OR Kirgizstan[TIAB] OR Kirghizia[TIAB] OR Kirgizia[TIAB] OR Kyrgyz[TIAB] OR Kirghiz[TIAB] OR Kyrgyz Republic[TIAB] OR Lao[TIAB] OR Laos[TIAB] OR Latvia[TIAB] OR Lebanon[TIAB] OR Lesotho[TIAB] OR Basutoland[TIAB] OR Liberia[TIAB] OR Libya[TIAB] OR Lithuania[TIAB] OR Macedonia[TIAB] OR Madagascar[TIAB] OR Malagasy Republic[TIAB] OR Malawi[TIAB] OR Nyasaland[TIAB] OR Malaysia[TIAB] OR Malaya[TIAB] OR Malay[TIAB] OR Maldives[TIAB] OR Mali[TIAB] OR Marshall Islands[TIAB] OR Mauritania[TIAB] OR Mauritius[TIAB] OR Mayotte[TIAB] OR Mexico[TIAB] OR Micronesia[TIAB] OR Moldova[TIAB] OR Moldovia[TIAB] OR Mongolia[TIAB] OR Montenegro[TIAB] OR Morocco[TIAB] OR Mozambique[TIAB] OR Myanmar[TIAB] OR Burma[TIAB] OR Namibia[TIAB] OR Nepal[TIAB] OR Nicaragua[TIAB] OR Niger[TIAB] OR Nigeria[TIAB] OR Pakistan[TIAB] OR Palau[TIAB] OR Palestine[TIAB] OR Panama[TIAB] OR Papua New Guinea[TIAB] OR Paraguay[TIAB] OR Peru[TIAB] OR Philippines[TIAB] OR Romania[TIAB] OR Rumania[TIAB] OR Roumania[TIAB] OR Russia[TIAB] OR Russian Federation[TIAB] OR USSR[TIAB] OR Soviet Union[TIAB] OR Union of Soviet Socialist Republics[TIAB] OR Rwanda[TIAB] OR Ruanda-Urundi[TIAB] OR Samoa[TIAB] OR Samoan Islands[TIAB] OR Sao Tome[TIAB] OR Principe[TIAB] OR Senegal[TIAB] OR Serbia[TIAB] OR Montenegro[TIAB] OR Yugoslavia[TIAB] OR Seychelles[TIAB] OR Sierra Leone[TIAB] OR Solomon Islands[TIAB] OR Somalia[TIAB] OR South Africa[TIAB] OR Sri Lanka[TIAB] OR Ceylon[TIAB] OR Saint Kitts[TIAB] OR St Kitts[TIAB] OR Saint Christopher Island[TIAB] OR Nevis[TIAB] OR Saint Lucia[TIAB] OR St Lucia[TIAB] OR Saint Vincent[TIAB] OR St Vincent[TIAB] OR Grenadines[TIAB] OR Sudan[TIAB] OR Suriname[TIAB] OR Surinam[TIAB] OR Swaziland[TIAB] OR Syria[TIAB] OR Syrian Arab Republic[TIAB] OR Tajikistan[TIAB] OR Tadzhikistan[TIAB] OR Tadjikistan[TIAB] OR Tanzania[TIAB] OR Thailand[TIAB] OR Timor-Leste[TIAB] OR East Timor[TIAB] OR Togo[TIAB] OR Togolese Republic[TIAB] OR Tonga[TIAB] OR Tunisia[TIAB] OR Turkey[TIAB] OR Turkmenistan[TIAB] OR Turkmenia[TIAB] OR Tuvalu[TIAB] OR Uganda[TIAB] OR Ukraine[TIAB] OR Uruguay[TIAB] OR Uzbekistan[TIAB] OR Vanuatu[TIAB] OR New Hebrides[TIAB] OR Venezuela[TIAB] OR Vietnam[TIAB] OR Viet Nam[TIAB] OR West Bank[TIAB] OR Gaza[TIAB] OR Yemen[TIAB] OR Zambia[TIAB] OR Zimbabwe[TIAB] OR Rhodesia[TIAB] |

| DISABILITY: 1. Medline | |
|---|---|
| MeSH | Disabled person |
| Other | OR disabled person* OR person with disabilit* OR persons with disabilit* OR people with disability* OR handicapped person* OR handicapped people |
| Other | Physical impair*or physically impair* OR physical deficien* OR physically deficien* OR physical disab*OR physically disab* OR physical handicap* OR physically handicap* OR physically challeng* |
| MeSH | Cerebral palsy (mh) OR Musculoskeletal Abnormalities [MH] |
| Other | OR Cerebral pals* OR spinal dysraphism OR Spina bifida OR muscular dystrophies OR Muscular dystroph* OR Arthritis OR Arthriti* OR Osteogenesis imperfecta OR Musculoskeletal abnormalit* OR Musculo-skeletal abnormalit* OR Muscular abnormalit* OR Skeletal abnormalit* OR limb abnormalit* OR Chronic Brain Injury OR Amputation* or Amputee OR Clubfoot OR Poliomyelitis OR Polio* OR Paraplegia OR Paraplegi* OR Paralys* OR Paralyz* OR Hemiplegia OR Hemiplegi* |
| MeSH | Hearing loss[MH] |
| Other | OR Hearing loss* OR hearing impair* OR hearing deficien* OR hearing disable* OR hearing disabili* OR hearing handicap* OR acoustic loss*OR acoustic impair* OR acoustic deficien* OR acoustic disable* OR acoustic disabili* OR acoustic handicap* OR Deaf* or hearing loss |
| MeSH | Blindness[MH] |
| Other | vision loss* OR vision impair* OR vision deficien* OR vision disable* OR vision disabili* OR vision handicap* OR visual loss* OR visual impair* OR visually impair* OR visual deficien* OR visually deficien* OR visual disable* OR visually disable* OR visual disabili* OR visually disabili* OR visual handicap* OR visually handicap* OR low vision OR reduced vision OR (blind* NOT double blind* NOT blinding NOT triple blind*) |
| MeSH | schizophrenia and disorders with psychotic features[MH] OR |
| Other | Mental disorder* OR Schizophreni* OR Psychosis OR psychoses OR Psychotic Disorder* OR Schizoaffective Disorder* OR Schizophreniform Disorder* OR Dementia* OR Alzheimer* |
| Other | intellectual illness* OR intellectual impair* OR intellectual deficien* OR intellectual disable* OR intellectual disabili* OR intellectual handicap* OR intellectual retard* OR mental ill OR mentally ill OR mental illness* OR mental impair* OR mentally impair* OR mental deficien* OR mentally deficien* OR mental disable* OR mentally disable* OR mental disabili* OR mental handicap* OR mentally handicap* OR developmental impair* OR developmentally impair* OR developmental deficien* OR developmentally deficien* OR developmental disable* OR developmentally disable* OR developmental disabili* OR developmentally disabili* OR developmental handicap* OR developmentally handicap* OR developmental retard* OR developmentally retard* OR psychological ill OR psychologically ill OR psychological illness* OR psychological impair* OR psychologically impair* OR psychological deficien* OR psychologically deficien* OR psychological disable* OR psychologically disable* OR psychological disabili* OR psychological handicap* OR psychologically handicap* |
| MeSH | Learning disorders[MH] |
| Other | OR learning disorder* OR communication disorders OR communication disorder* OR language disorder* OR speech disorder* OR speech disorder* |
| MeSH | Pervasive Child Development Disorders[MH] |
| Other | OR autistic OR autism OR asperger* or dyslexi* OR Down's Syndrome OR Down Syndrome OR Mongolism or Trisomy 21 |

| MATERNAL HEALTH: 1. Medline | |
|---|---|
| MESH General Maternal Health | Pregnancy [Mesh] OR pregnancy outcome* [Mesh] OR pregnancy complication* [Mesh] OR unplanned pregnancy [Mesh] OR unwanted pregnancy [Mesh] OR adolescent pregnancy [Mesh] OR maternal welfare [Mesh] OR maternal health [Mesh] OR obstetric* [Mesh] |
| General Maternal Health | (Pregnan* OR pregnancy outcome* OR pregnancy complication* OR unplanned pregnancy OR unwanted pregnancy OR adolescent pregnancy OR maternal welfare OR maternal health OR obstetric* OR safe motherhood OR maternal health service* OR obstetric procedure* OR obstetric care OR obstetric surgery OR emergency obstetric care OR EmOC OR fetus OR intrauterine pregnancy OR IUP OR unborn child).ti,ab |
| MESH Antenatal (inc. prenatal) | Prenatal care [Mesh] OR prenatal diagnosis [Mesh] OR prenatal injuries [Mesh] OR spontaneous abortion [Mesh] |
| Antenatal (inc. prenatal) | OR (prenatal* OR prenatal care OR prenatal health OR prenatal service* OR prenatal diagnosis OR pregnancy test* OR antenatal* OR antenatal care OR antenatal health OR antenatal serivice* OR antenatal screening OR fetal ultrasound OR miscarriage OR fetal loss OR pregnancy loss OR spontaneous abortion OR fetal therapies OR fetal monitoring).ti,ab |
| MESH Intrapartum (+ 'perinatal') | Partuition [Mesh] OR perinatal Care [Mesh] OR delivery, obstetric [Mesh] |
| Intrapartum (+ 'perinatal') | OR (birth OR childbirth OR intrapartum OR parturition OR perinatal* OR perinatal care OR perinatal health OR perinatal service* OR labor OR labor complications OR labor pain OR delivery OR safe delivery OR skilled birth attend* OR stillbirth OR induction of labour OR caesarian section OR C-section)ti.ab |
| MESH Postnatal | Postnatal care [Mesh] OR postpartum period [Mesh] |
| Postnatal | OR (postpartum OR postpartum period OR postbirth OR postnatal* OR postnatal care OR postnatal health OR postnatal service* OR newborn care OR infant OR neonat* OR neonatal health OR infant health OR infant welfare OR newborn health OR mother and baby OR baby health OR puerperium OR postpartum contraception).ti,ab |

| REPRODUCTIVE HEALTH: 1. Medline | |
|---|---|
| MESH General RH | Reproductive health [Mesh] OR reproductive medicine [Mesh] |
| Other | OR (reproductive health OR reproductive medicine OR reproductive health service* OR minimum initial service package).ti,ab |
| MESH Urogenital | Female urogenital disease [Mesh] OR Male urogenital disease [Mesh] OR uterine cervical neoplasms [Mesh] |
| Other | OR (genital trauma OR genital injury OR trophoblastic disease OR choriocarcinoma OR human papillomavirus OR HPV OR cervical cancer* OR cervical tumor* OR cervical neoplasm* OR cervical malignan* OR cancer of cervix OR cervical smear OR cervical screening).ti,ab |
| MESH menstruation | Menstruation [Mesh] OR Menstruation disturbances [Mesh] |
| Other | OR (Menstrua* OR menstrual health OR menstrual period OR menstrual education OR period education OR menstrual hygiene OR menstrual cup OR menstrual flow OR menstrual supression Or menstrual regulation OR sanitary pad OR tampon OR menses OR menarche).ti,ab |

| STIS: 1. Medline | |
|---|---|
| MESH Genral STIs | Sexually transmitted diseases [Mesh] |
| Other | OR (Sexually transmitted disease* OR STD* OR sexually transmitted infection* OR STI*).ti,ab |
| MESH HIV/AIDS | HIV [Mesh] |
| Other | OR (Human immunodeficiency virus OR HIV OR acquired immunodeficiency syndrome OR acquired immune deficiency syndrome OR AIDS).ti,ab |
| MESH PMTCT | |
| Other | (Vertical transmission OR mother-to-child transmission OR mother to child transmission OR MTCT OR mother-to-baby transmission OR mother to baby transmission OR prevention of mother-to-child transmission OR prevention of mother to child transmission OR PMTCT).ti,ab |
| MESH Testing | |
| Other | (STI testing OR testing for sexually transmitted infections OR STD testing OR testing for sexually transmitted diseases OR VC OR voluntary counselling OR voluntary counselling and testing OR confidential testing OR test kit).ti,ab |
| MESH Treatment | |
| Other | (STI treatment OR STD treatment OR treating STIs OR treating STDs OR treating sexually transmitted infection* OR treating sexually transmitted disease* OR contact tracing OR antiretroviral* OR ART OR antiretroviral therapy).ti,ab |

| CSE: 1. Medline | |
|---|---|
| MeSH Adolescent Health Terms | Pregnancy in adolescence (MeSH) |
| Adolescent Health Other Terms | adolescent sexual health OR adolescent reproductive health OR youth sexual health OR adolescent reproductive health OR youth reproductive health OR adolescent health OR youth health OR adolescent health services OR youth friendly services OR adolescent friendly services OR youth program* OR pregnant adolescents OR teenage* pregnancy |
| CSE MeSH Terms | Sex education (MeSH) |
| CSE Other Terms | Comprehensive sexuality education OR sex education OR sex ed OR sex education program* OR sexual health education OR sexuality education OR (pregnancy AND prevent*) OR life skills OR family life education OR holistic sexuality education OR HIV education OR prevention education OR relationship and sexuality education OR sex counselling OR health education OR ABC OR abstinence education OR abstinance based education OR evidence based sex education OR reality based sexuality education OR family planning education |
| Intervention MESH Terms | School health service (MESH). But didn't explode |
| Intervention Terms | school based intervention OR school health services OR community based intervention |
| Peer Education Terms | peer education OR peer led intervention OR peer mentoring OR mentors OR student led OR peer to peer |

| FAMILY PLANNING AND CONTRACEPTION: 1. Medline | |
|---|---|
| Contraception (mesh) | family planning services (mesh) OR contraception (mesh) OR contraception, barrier (mesh) OR long-acting reversible contraception (mesh) OR condoms (mesh) OR intrauterine devices (mesh) OR family planning programs (mesh) OR contraceptive behavior (mesh) |
| Contraception (other) | family planning OR reproductive plan* OR contracepti* OR birth control OR birth spacing OR child spacing OR condom* OR the pill OR oral contracepti* OR microbicide OR diaphragm OR IUD OR intrauterine device OR contraceptive implant OR progestogen only contraceptive OR natural family planning OR lactational amenorrhea OR LAM or postpartum amenorrhea OR post-partum amenorrhea OR period abstinence OR rhythm method OR calendar method OR sexual abstinence OR family planning program OR population control OR contracepti* device* OR contracepti* agent* OR pregnancy prevent* OR fertility control OR family planning service* OR family planning clinic* |
| Emergency contraception (mesh) | contraception, postcoital (mesh) |
| Emergency contraception (other) | emergency contracepti* OR morning after pill OR emergency contracepti* pill OR emergency postcoit* contracepti* OR postcoit* contracepti* OR morning after contracepti* OR day after contracepti* OR ECP OR advance* provision OR self administr* |
| Infertility (mesh) | infertility (mesh) OR fertilization in vitro (mesh) OR reproductive techniques, assisted (mesh) |
| Infertility (other) | infertil* OR subfertil* OR assisted reproduction OR assited reproducti* techniques OR ART OR IVF OR in vitro fertili?ation OR in-vitro fertili?ation |

| ABORTION: 1. Medline | |
|---|---|
| Medical abortion (mesh) | abortion, induced (mesh) |
| Medical abortion (other) | abortion OR induced abortion OR termination of pregnanc* OR pregnancy termination OR medic* abortion OR menstrual regulation OR unsafe abortion OR abortion pill OR medical termination of pregnancy |
| Surgical abortion (mesh) | dilatation and curettage (mesh) OR vacuum curettage (mesh) |
| Surgical abortion (other) | surgical abortion OR dilatation and cutterage OR D&C OR vacuum cutterage OR vacuum aspiration OR cutterage OR surgical termination of pregnancy OR aspiration abortion or unsafe abortion |
| Miscarriage (mesh) | abortion, spontaneous (mesh) |
| Miscarriage (other) | miscarriage* OR spontaneous abortion OR pregnancy loss OR spontaneous pregnancy loss OR fetal death OR recurrent pregnancy loss OR early pregnancy loss |
| Abortion complications (mesh) | Abortion, incomplete (mesh) OR abortion, septic (mesh) |
| Abortion complications (mesh) | abortion, incomplete (mesh) OR abortion, septic (mesh) |
| Abortion complications (other) | incomplete abortion OR abortion complication* OR unsafe abortion OR uterine infection OR uterine perforation OR abortion-related complications OR post abortion care OR post-abortion care |

| SEXUAL VIOLENCE: 1. Medline | |
|---|---|
| GBV MESH | gender-based violence (mesh) OR sexual violence (mesh) OR rape (mesh) OR domestic violence (mesh) OR intimate partner violence (mesh) OR sexual harassment (mesh) OR battered women (mesh) |
| GBV | partner violence OR family violence OR violence against women OR domestic violence OR dating violence OR family violence OR sexual violence OR physical violence OR rape OR intimate partner violence OR domestic violence<br>OR sexual abuse OR partner abuse OR intimate partner abuse OR spousal abuse OR spouse abuse OR domestic abuse OR wife abuse<br>OR sex crime OR sexual crime<br>OR assault OR physical assault OR sexual assault OR sexual harassment OR sexual coercion OR forced sex OR sexual slavery<br>OR abused woman OR abused women OR battered woman OR battered women OR woman, abused OR woman, battered OR women, abused OR women, battered OR spous* abuse OR battered wom*n |
| FGM MESH | “Circumcision, Female” [Mesh] |
| FGM | Female Circumcisions OR Female Circumcision OR Infibulation OR Infibulations OR Clitoridectomy OR Clitoridectomies OR Clitorectomy OR Clitorectomies OR Female Genital Cutting OR Female Genital Mutilation OR Female Genital Mutilations |

| SEXUALITY, SEXUAL HEALTH, AND RIGHTS: 1. Medline | |
|---|---|
| General. MESH | Reproductive rights (MESH) OR gender identity (MESH) OR Sexual and Gender minorities (MESH) OR sex worker (MESH) OR sexual partner (MESH) |
| General | reproduction rights OR sexuality OR sexual behaviour OR gender identity OR sexual minorit* OR sex worker OR sex* partner* OR sex* counselling OR sexual freedom |
| Sexuality MESH | sexual health (MESH) OR psychosexual development (MESH) OR physiological sexual dysfunction* OR psychological sexual dysfunction* OR sexuality (MESH) OR sexology (MESH) OR Reproduction, asexual OR sexual abstinence (MESH) |
| Sexuality | Libido OR libido disorder* OR sexology OR sexual health OR psychosexual development OR sex development OR stimulation OR sexual satisfaction* OR sexual pleasure OR orgasm* OR ejaculation OR sexual dysfunction* OR physiological sexual dysfunction* OR psychological sexual dysfunction* OR impotence OR erectile dysfunction OR premature ejaculation OR dyspareunia OR painful intercourse OR anorgasmia OR Orgasm OR self pleasuring OR masturbation OR sexual arousal OR sexual fantasy satisf* sex OR satisfacation with sex OR sexual abstinence |
